# Current Approaches and Techniques in Physiologically Based Pharmacokinetic (PBPK) Modelling of Nanomaterials

**DOI:** 10.3390/nano10071267

**Published:** 2020-06-29

**Authors:** Wells Utembe, Harvey Clewell, Natasha Sanabria, Philip Doganis, Mary Gulumian

**Affiliations:** 1 National Institute for Occupational Health, P.O. Box 4788, Johannesburg 2000, South Africa; WellsU@nioh.ac.za (W.U.); NatashaS@nioh.ac.za (N.S.); 2 Ramboll US Corporation, Research Triangle Park, NC 27709, USA; hclewell@ramboll.com; 3 School of Chemical Engineering, National Technical University of Athens, Zografou Campus, 15780 Athens, Greece; fdoganis@chemeng.ntua.gr; 4 Hematology and Molecular Medicine, University of the Witwatersrand, Johannesburg 2000, South Africa

**Keywords:** nanomaterial, PBPK, absorption, distribution, metabolism, elimination, hazard, risk

## Abstract

There have been efforts to develop physiologically based pharmacokinetic (PBPK) models for nanomaterials (NMs). Since NMs have quite different kinetic behaviors, the applicability of the approaches and techniques that are utilized in current PBPK models for NMs is warranted. Most PBPK models simulate a size-independent endocytosis from tissues or blood. In the lungs, dosimetry and the air-liquid interface (ALI) models have sometimes been used to estimate NM deposition and translocation into the circulatory system. In the gastrointestinal (GI) tract, kinetics data are needed for mechanistic understanding of NM behavior as well as their absorption through GI mucus and their subsequent hepatobiliary excretion into feces. Following absorption, permeability (*P_t_*) and partition coefficients (*PCs*) are needed to simulate partitioning from the circulatory system into various organs. Furthermore, mechanistic modelling of organ- and species-specific NM corona formation is in its infancy. More recently, some PBPK models have included the mononuclear phagocyte system (MPS). Most notably, dissolution, a key elimination process for NMs, is only empirically added in some PBPK models. Nevertheless, despite the many challenges still present, there have been great advances in the development and application of PBPK models for hazard assessment and risk assessment of NMs.

## 1. Introduction

Physiologically-based pharmacokinetic (PBPK) modelling is a computational approach that simulates the absorption, distribution, metabolism and elimination (ADME) of chemical substances in the bodies of organisms. PBPK models consist of systems of differential mass balance equations representing biological tissues and fluids as well as physiological processes. PBPK models are useful for predicting internal doses that can be used to replace administered or applied dose in the derivation of dose-response relationships. Dose-response relationships have been reported to be accurate when expressed on the basis of the internal dose [1] which can ultimately be used to derive points-of departure such as the no-observed-adverse-effect level (NOAEL), the lowest-observed-adverse-effect level (LOAEL), the benchmark dose (BMD) and benchmark concentration (BMC). Other applications of PBPK modelling in risk assessment include interspecies extrapolation of the dose-response relationship (based on estimates of the internal dose), route-to-route extrapolation, estimation of response from varying exposure conditions, estimation of human variability (within the whole population or subpopulations), as well as high-to-low dose extrapolation [1]. PBPK modelling is increasingly being utilized in conjunction with in vitro *to* in vivo *extrapolation* (IVIVE) methods to predict in vivo clearance from in vitro metabolic data [2,3].

Development of PBPK models requires species-specific physiological and anatomical data as well as substance-specific pharmacokinetic data and partition coefficients of the compound in various tissues [4]. Using these parameters, PBPK models have been developed for many conventional substances including organic compounds and other inorganic substances. However, as there are major differences between the ADME behaviors of nanomaterials (NMs) and those of conventional chemical compounds, some additional factors should be considered in the development of PBPK models for NMs [5]. These additional factors have been included in the development of various PBPK models for inorganic NMs such as quantum dots (QDs) [6,7,8], carbon based NMs [9], metal based NMs including silver (Ag) [10,11,12], gold (Au) [13,14,15,16], metal oxides such as titanium dioxide (TiO_2_) [17,18,19,20], cerium dioxide (CeO_2_) [16,21,22] and zinc oxide (ZnO) [23]. PBPK models have also been developed for polymeric NMs such as polyacrylamide (PAA) [24], poly(lactic-co-glycolic) acid (PLGA) [25], the anticancer agent SNX-2112 [26] and other polymers [27,28]. Differences exist in the approaches and techniques for developing these PBPK models. For example, some of the models include dissolution as one of the elimination processes [10,11], while others do not [6,7]. This paper aims to present an overview of the challenges in the PBPK modelling of NMs as well as a critical review of the various approaches that are utilized in the PBPK modelling of NMs. The paper will outline areas of concordance and divergence and hence point out future directions in the PBPK modelling of NMs.

## 2. General Approaches in PBPK Modelling of Nanomaterials

### 2.1. Top-Down and Bottom-Up as Well as Deterministic and Probabilistic Approaches

Pharmacokinetic models, including PBPK models, can be built based mainly on the observed experimental data (‘top down’ approach) or based on our broader understanding of the human body and its mechanisms (‘bottom up’). ‘Top-down’ approaches utilize experimental pharmacokinetic data to develop a PBPK model empirically, where both the structure and the parameters are derived from the experimental data. The disadvantage of this approach is the limited scope emanating from use of empirical data that is only relevant to the range of the input data [29]. In that regard, when there are changes in the species or exposure conditions, the model may no longer be applicable.

The ‘bottom-up’ approach, on the other hand, integrates a large number of chemical-specific data, physiological or anatomical parameters as well as pharmacokinetic processes. The structure of ‘bottom-up’ PBPK models explicitly represents the current understanding of the underlying physical, chemical, and/or biological processes [30,31,32]. The mechanistic ‘bottom-up’ approach mathematically describes the relationship between variables based on causative underlying principles [33]. The disadvantage of this approach is that often, as in the case for NMs, the processes that affect the pharmacokinetic or toxicokinetic behavior of a NM may not be fully understood.

Often top-down and bottom up approaches are combined in a ‘middle-out’-approach to allow the utilization of available in vivo information as well as the determination of unknown or uncertain parameters. In this regard, parameters with unknown values are optimized by fitting the unknown model parameters against the experimental data as has been performed in PBPK models for some NMs [13,24,34]. In one such example, Li et al. [25] utilized multivariate regression to ascertain relationships between physicochemical properties and distribution parameters. This middle-out (semi-mechanistic) model was subsequently used to predict the distribution of polyethylene glycol (PEG)-coated polyacrylamide nanoparticles.

Predictive models may be classified as deterministic or probabilistic (stochastic) depending on the nature of the input variables. Deterministic models utilize fixed values of the input variables, while probabilistic models can take into account the uncertainty and variability in one or more of the input parameters [33]. Deterministic modeling does not represent the variability in exposure and physiological/biochemical characteristics that is present in any population, while probabilistic modeling utilizes probability distributions in the inputs rather than single-point estimates and thereby characterizes the potential uncertainty and variability in predicted pharmacokinetics in a population [35]. Through the iterative generation of random values from input parameter distributions in simulation runs that can number in the thousands, probabilistic risk assessment allows numerous outputs that represent the possible differences within a target population [36]. Therefore, the potential impact of various sources of uncertainty and variability may be characterized, such as human inter-individual variability in pharmacokinetics [37,38], pharmacodynamics [39] and genetics [40].

Parameters for PBPK models are sometimes estimated by “fitting” the model to informative experimental data; that is, by minimizing the negative log likelihood of the parameters based on a comparison of predicted and observed values. This fitting can be implemented in either a frequentist or “classical” approach or in a Bayesian approach. In a frequentist approach, the probability is usually assessed based on minimizing some form of “sum of squares” residual between the predicted and observed data [41]. Since such frequentist analyses tend to ignore results from previous studies, modelling based on this approach may produce unrealistic results [42]. On the other hand, Bayesian analysis permits the use of available prior information [43,44]. The basic tool of a Bayesian analysis is Bayes’ theorem, which dictates how to update prior distributions describing the investigator’s belief based on observations or assumptions. The application of Bayesian approaches in PBPK modelling of NMs appear to be rare. In that regard, Cheng et al. [45] developed and implemented a Bayesian-based PBPK model for a probabilistic risk assessment of Au NPs, while Pery et al. [9] used the approach in the PBPK model for 99m-Technetium-labelled carbon NPs.

Uncertainties may also be reduced by developing PBPK models that integrate physiological and enzymatic changes that accompany specific conditions such as pregnancy [46,47,48] and childhood (pediatric) development [49]. However, there appears to be no PBPK models that have been developed for these specific situations, although it has been shown, for example, that NMs may cross the placenta and reach the fetus [50,51,52].

### 2.2. Transport and Permeation Processes—Perfusion-Limited versus Permeability-Limited

The transportation and permeation of substances from the blood to the tissue compartments may be described as either perfusion-limited (blood-flow-limited) or permeability-limited (also termed diffusion-limited). In perfusion-limited PBPK models, the concentration of the substance in tissues is assumed to attain fast equilibrium with the substance in the circulation system, an indication that the substance can easily penetrate tissue cell membranes [53]. Therefore, in perfusion-limited models, the transportation rate of the NPs from blood into tissues depends only on blood flow-rates [25] and penetration of the substance into tissues is only limited by blood perfusion into the tissues. Perfusion-limited kinetics “tends to occur in small lipophilic molecules where the blood flow to the tissue becomes the limiting process” [54]. Perfusion-limited models can either be based an assumption of a well-stirred compartment, where there is no concentration gradient or on a dispersion model, in which concentration gradients exist although a diffusion barrier cannot be identified [55]. The steady-state tissue concentration in either a perfusion- or permeability-limited description for chemical compounds is determined by thermodynamic partitioning based on the relative chemical activity in the blood and tissue matrix. These partition coefficients (PCs) can be estimated from in vitro or in vivo distribution data (ratio of tissue and plasma concentrations at steady-state) and can often be estimated by quantitative structure/property relationship (QSPR) modelling.

Permeability-limited PBPK models are typically developed for larger polar molecules, where distribution of the substance into the tissue is limited by the permeability of the cell membrane. In these models, each tissue is divided into two compartments that represent the intracellular space and the extracellular space, separated by a cell membrane barrier [53,54]. The intracellular space and the extracellular space should be described separately [56]. Although the concentration of the substances will also reach equilibrium in permeability-limited models, the time to reach equilibrium is highly dependent on the substance-specific permeability rather than on the blood flow [54].

Perfusion-limited models have much less biophysical detail than the two-sub-compartment permeability-limited models, which have both vascular and extravascular tissue space [57]. Tissue uptake in perfusion-limited models is calculated by the general mass balance equation presented in Equation (1), where Q_t_ is the blood flow to tissue, PC is the partition coefficient., V_t_ is the tissue volume and blood is the concentration of the NM in arterial blood:

(1) $$\frac{{\mathrm{dC}}_{\mathrm{tissue}}}{\mathrm{dt}}=\frac{Q_{t}}{V_{t}}\left(C_{\mathrm{blood}}-\frac{C_{\mathrm{tissue}}}{\mathrm{PC}}\right).$$

Tissue uptake in permeability-limited models is calculated by two general mass balance equations (Equations (2) and (3)), which, in addition to V_t_ and PC, have P_t_, the permeability of the tissue:

(2) $$\frac{{\mathrm{dC}}_{\mathrm{tissue}}}{\mathrm{dt}}=\frac{Q_{t}}{V_{t}}\left(C_{\mathrm{blood}}-C_{\mathrm{vascular}}\right)-\frac{P_{t}}{V_{t}}\left(C_{\mathrm{tissue}}-\frac{C_{\mathrm{extravascular}}}{\mathrm{PC}}\right)$$

(3) $$\frac{{\mathrm{dC}}_{\mathrm{extravascular}}}{\mathrm{dt}}=\frac{P_{t}}{V_{t}}\left(C_{\mathrm{tissue}}-\frac{C_{\mathrm{extravascular}}}{\mathrm{PC}}\right).$$

The rate of uptake and efflux of NMs in a tissue in perfusion-limited models is mainly determined by blood flow and PCs only (Equation (1)), whereas it is also controlled by P_t_ in a permeability-limited model. While the permeability-limited model is more biophysically realistic, it is more computationally demanding and expensive [57].

Both perfusion- and permeability-limited PBPK models have been developed for NMs. For example, Chen et al. [23] applied a perfusion-limited PBPK model to describe the toxicokinetics of 10 and 71 nm ZnO NPs. While the simulation of Zn(NO_3_)_2_ fitted the experimental data, the simulation of ZnO NPs only improved after replacing PCs of the NPs with those of Zn(NO_3_)_2_ after day number 7. In addition, while, according to the model developers, the dissolution of ZnO NPs after day 7 could explain this observation, permeability of the tissues could also be the reason. A perfusion-limited model would suit the ionic Zn(NO_3_)_2_ and not the ZnO NPs which would be hindered by the membrane until after the reported day 7 when most of the NPs may have undergone substantial dissolution. Indeed, a life-time of 90 min has been reported for ZnO NPs in aqueous media [58], while 70% of Zn from Zn NPs was reported to have undergone dissolution to form secondary ZnS particles after 10 days in the aqueous environment of a wastewater treatment plant [59]. For a similar reason, a perfusion-limited PBPK model predicted very well the experimentally observed bio-persistence of QDs but not the early-time QD biodistribution [7]. Furthermore, although both a perfusion-limited model and a permeability-limited model adequately simulated the pharmacokinetics of 100 nm PEG-coated AuNPs, the permeability-limited model provided a better simulation for the 13 nm Au NPs [14]. A permeability-limited PBPK model also provided better simulation of the biodistribution of PLGA NPs (57.5–133.5 nm) than the perfusion-limited model [25].

Some authors also report to have utilized perfusion-limited processes for some organs and permeability-limited processes for other organs in the same model. For example, the models by Carlander et al. [22] as well as Li et al. [24] used both perfusion-limited and permeability-limited processes. The former model had better predictive capability for 5 nm than for 30 nm CeO_2_ NPs, while the latter model adequately described the biokinetics of 35 nm PAA NMs. Perfusion-limited and permeability-limited processes were also utilized by Li et al. [21] to adequately describe the biodistribution of both small (25 nm) and large (90 nm) CeO_2_ NPs. Therefore, there is need for more studies on the impact of transport process (perfusion-limited and permeability-limited) on the predictive capability of models in relation to composition, particle size and other physicochemical properties. Specifically, it is important to ascertain the size ranges, NM composition, functionalization and charges that are suitable for each approach, that is perfusion-limited and permeability-limited models.

## 3. Pharmacokinetic Modelling

### 3.1. Absorption

Absorption has been defined as the process by which NMs are transported from the external site of exposure into an internal biological space [5]. Following administration, NMs are prone to either pre-absorption clearance or absorption, where the former involves degradation and direct removal from the administration site through such processes as exhalation or mucociliary clearance, while those that evade pre-absorption clearance can be absorbed into the blood and lymphatic systems after crossing the mucus and lung epithelium cells [53]. The amount of NMs deposited in the lung is estimated using lung dosimetry models such as the International Commission on Radiological Protection (ICRP) Human Respiratory Tract Model [11] or the Multiple-Path Particle Dosimetry (MPPD) model [21]. NMs that are administered through non-intravenous injection (i.e., Intraperitoneal, intradermal, subcutaneous, intramuscular) need to be absorbed into the circulatory system prior to distribution into other organs and tissues [5]. These NMs are primarily absorbed through lymph vessels or macrophages and further cell-trafficked into regional lymph nodes [53,60].

Following dermal administration, several factors, including skin thickness, skin humidity, temperature, barrier integrity, mechanical flexion, NM properties, contaminants in NMs and dissolution of NPs may increase their dermal uptake. Absorption through intact skin has been observed to occur for NMs smaller than 4 nm, while penetration of NMs larger than 45 nm may only take place in severely damaged skin [61]. Orally administered NMs interact with the local environment in the gastrointestinal tract, where they are surrounded by a corona of proteins and biomolecules from food [62]. Some NMs may be trapped in mucus and expelled through feces, while those that migrate through the mucus have to pass through the epithelium to reach the systemic circulation [63] through transcytosis via specialized intestinal epithelial cells, the M cells, as well as uptake via intestinal lymphoid tissues, the Peyer’s patches [64].

In comparison to the case with chemical compounds, where uptake occurs largely through passive diffusion [65], NMs may enter cells through active transport pathways, which largely involve size- and surface-chemistry-dependent endocytosis. Endocytosis is an inclusive term that represents different cellular uptake mechanisms of NMs, including phagocytosis, as well as receptor-independent (macropinocytosis) and receptor-mediated endocytosis [66]. For this reason, PBPK models for conventional molecules are generally not suitable for modelling the absorption of NMs [67].

In PBPK modelling of NMs, two general uptake pathways can be identified based on location—Uptake directly from the blood to endothelial cells (or macrophages) in the tissue capillary blood vessels and permeation into tissue, depending on the relative size of the NM and pores of capillary walls, followed by uptake by tissue macrophages [56,66].

Indeed, endocytosis (cell uptake) of NMs is a very complex process that depends on host-specific and environmental factors such as cell type and serum proteins [68,69,70], as well as NM-specific factors such as size, shape, functionalization and charge [71,72,73]. Furthermore, at very high concentrations, colligative behaviors that depend on number density, possibly due to masking of cell surfaces or obstruction of cellular mechanisms, have been reported for some NMs [74]. Therefore, simulation of endocytosis of NMs increases the complexity and uncertainty of a PBPK model. To avoid this complexity, most PBPK models simulate a size-independent endocytosis of NMs using a linear equation, assuming either uptake from the tissue [24] or from the blood [11,17]. For example, Bachler et al. [11] have based uptake rates on organ-specific characteristics including capillary wall type, phagocytosis efficiency as well as the amount of Ag NPs that passes through the capillary walls of each organ as determined from organ blood flow normalized to the total blood volume. Lin et al. [56] found it more convenient and appropriate to describe uptake of 13 nm Au NPs through endocytosis from tissue and uptake of 100 nm Au NPs from blood.

The uptake rate has been observed to become slower as saturation is approached [75]. For that reason, some authors utilized a time-dependent uptake rate that is a function of time as described by the Hill equation [56] or the Michaelis-Menten equation [15]. Indeed, Lin et al. [56] obtained more accurate predictions of endocytosis of NMs using the Hill function than through the use of linear equations, since the Hill equation is more suitable for saturable processes such as uptake of NMs. On the other hand, some authors have utilized time-independent uptake rates [11,17], that is, without saturation. For example, Li et al. [24] used the same maximum uptake rate for all compartments, except for the spleen because of its mesh-like structure which could trap NMs and thereby delay their contact with phagocytizing cells.

The cellular uptake of NMs may also be dependent on the solubility/dissolution rates of the NMs. For example, cellular uptake and intracellular kinetics for insoluble to moderately water-soluble nickel microparticles, such as crystalline Ni_3_S and crystalline nickel sulfide, were reported to be different from those of highly water-soluble nickel species such as NiCl_2_.6H_2_O and NiSO_4_.6H_2_O [76]. Consequently, exposure at the same concentration of a highly soluble NM or a less soluble NM would result different doses of NMs delivered to cells. Although the model was for microparticles, the same principles will apply for PBPK models for NMs.

Although some research seems to indicate that uptake is essentially an irreversible process, with particles accumulating in lysosomes [77], some PBPK models have included an organ release rate for NMs [11,56,78]. The uptake and organ release (exocytosis) rates are often obtained from the literature [56] as well as from direct in vivo measurements [15]. Unfortunately, there has been relatively much less investigation on exocytosis as compared to endocytosis [79]. Therefore, studies on exocytosis are necessary, particularly the rates of exocytosis of internalized NMs (especially from macrophages) in order to enable the evaluation of their chronic toxicity.

Silva et al. [78] could mathematically define organ release rate by Equations (4) and (5):

(4) $$\mathrm{organ}-\mathrm{release}-\mathrm{capillary}=k_{\mathrm{organ}-\mathrm{release}-\mathrm{cap}}{\;\times \;N}_{\mathrm{organ}-\mathrm{NPs}}$$

(5) $$\mathrm{organ}-\mathrm{release}-\mathrm{macrophages}={\;k}_{\mathrm{organ}-\mathrm{release}-\mathrm{macro}}{\;\times \;N}_{\mathrm{organ}-\mathrm{NPs}},$$

where k_organ-release-cap_ (min^−1^) and k_organ-release_macrophage_ (min^−1^) are constants that define the NM release from capillary and macrophages, while N_organ.NPs_ are the amount of NPs in the organ.

### 3.2. Distribution

Following intravenous administration, NMs enter the vascular system through which they are distributed to various organs and tissues of the body [80]. Following inhalation, NMs deposited in the alveolar region are engulfed by alveolar macrophages, which migrate to the tracheobronchial region for mucociliary clearance or to mediastinal lymph nodes [81,82]. Some NMs enter the epithelium by endocytosis [5]. More recently, the translocation kinetics of Au NPs across the lung epithelium was determined using alveolar epithelial cellular monolayers at the ALI [83]. Similarly, Kolanjiyil and Kleinstreuer [84,85] modelled the transport and deposition of inhaled NPs in the human respiratory system as well as transfer across barriers into systemic regions. Region-specific NP deposition results obtained from the model can be used to determine multi-compartmental parameters that can be useful in PBPK modelling.

NMs that reach the vascular system encounter blood cells, platelets, coagulation factors and plasma proteins. Adsorption of proteins on the surface of NMs results in the formation of a complex and dynamic corona which may affect uptake and distribution of NMs into various tissues [86]. These complex and dynamic processes would ideally need to be taken into account in PBPK models [22]. However, formation of corona is currently not a sufficiently understood process [62].

The vascular endothelium is a semi-selective barrier that regulates transport of the NMs from the vascular compartment to the extravascular space. Since the effective pore size of the endothelium is about 5 nm [87], NMs with size less than 5 nm achieve rapid equilibrium with the extravascular space, while larger particles undergo prolonged circulatory times because of slow transport across the endothelium [80].

In the vascular system, NMs are transported through three different patterns of flow, depending on the relative size of the blood vessel. In large vessels such as the aorta and human larger arteries, high Reynolds number flow prevails. In smaller vessels such as in arterioles, capillary and venules laminar flow is predominant, while single-file flow occurs in small capillaries [88]. Blood vessel endothelia are described as continuous, fenestrated or discontinuous, depending on the morphological features of the endothelium. Fenestrated endothelium exists in glands, digestive mucosa, kidneys and tumors, while discontinuous endothelium exists in the liver, spleen and bone marrow. Blood vessel endothelia influence the distribution of NMs into tissues, where NMs with less than 60 nm may have easier access into tissues with fenestrated or discontinuous endothelia [5]. Bachler et al. [11] categorized organs into four groups according to their capillary wall types:

- Non-sinusoidal non-fenestrated blood capillary type (brain, heart, lung, muscles)
- Non-sinusoidal fenestrated blood capillary type (intestines, kidneys, skin, testes)
- Sinusoidal blood capillary type with pores larger than 15 nm (liver, spleen)
- Myeloid bone marrow sinusoidal blood capillary type (bone marrow).

The model adequately described the levels for 15, 20, 60, 80 and 110 nm Ag NPs, indicating the applicability of the transfer functions based on the capillary wall type for determining the organ uptake rates. Bachler et al. [17] added an extra group of organs representing “all tissues and organs that are not covered by any other compartment, hence, could not be assigned to a specific group.” The model could adequately predict levels for 15 to 150 nm TiO_2_ NPs.

The density of open fenestrae appears to be an important factor in interspecies extrapolation. In that regard, Lin et al. [56] indicated that rats and pigs may be more suitable models than mice because, although the liver capillaries of mice, rats, pigs and humans can all be described as sinusoidal with open fenestrae, the average number of fenestrae per square micrometer in mice is much lower. The influence of blood vessels on the distribution of NMs is expressed as the permeability of the various organs.

#### 3.2.1. Permeability of Various Organs to Nanomaterials

The biodistribution of NMs into an organ or tissue requires the escape of the NMs from vascular flow, adherence to the vascular endothelium and migration across the endothelial cell (EC) barrier into the target tissue. This process is largely determined by the *P_t_* of the endothelial barrier to the NMs as well as the different physical attributes of the NMs [89]. Indeed, sensitivity analyses for some PBPK models have shown that *P_t_* values are among the most influential factors [22,56]. However, there is scarcity of information on the attributes that affect NM extravasation from vascular flow into various tissues because permeability assays are challenging to implement in vivo due to their complexity, high cost, heterogeneity, as well as large variability in results. For these reasons, there appear to be differences in the choice of this parameter in most PBPK models.

For example, Li et al. [24] used the same *P_t_* values for lungs, spleen, liver, kidneys, heart and bone marrow, while the *P_t_* for the brain compartment was set to zero, on the assumption of a highly efficient blood–brain barrier (BBB) for NMs. Li et al. [21] also assumed one *P_t_* for the brain, another value for the liver and the spleen and another *P_t_* for the other organs. Indeed, the BBB comprises of a specialized system of capillary endothelial cells that protect the brain from toxic substances including NMs. However, although the endothelial cells of the BBB lack fenestrations and endocytotic activity [90], NMs have often been detected in the brain probably through locations where this barrier is less well developed or damaged or through neuronal axons and dendrites [10,91]. Indeed, with regards to the latter pathway, NMs deposited in the nasal cavity have been shown to translocate through the sensory neuronal pathway to the olfactory bulb [92]. Despite these findings, this pathway is ignored in most PBPK models although it was included in the model by MacCalman et al. [93].

#### 3.2.2. Partition Coefficients

The propensity of a chemical to exit the blood compartment into an organ is measured by the *PC*. A *PC*, the ratio of the concentrations of a chemical in two phases in contact at equilibrium, characterizes the disposition of chemicals in living organisms [94]. Tissue-blood *PC*s are used in PBPK models to estimate the uptake and distribution kinetics of chemicals between blood and various organs. They provide the tendency of the substance to concentrate in a target tissue relative to its concentration in blood under equilibrium conditions [95]. Therefore, *PCs* provide an indication of the degree of accumulation of a substance in a tissue compared to another under steady-state conditions [96]. High *PC*s indicate that the NMs have “higher resident time within tissue than within blood, which leads to slower transportation rates from tissues back into blood circulation” [25].

For conventional substances, the octanol-water distribution coefficient (*K_ow_*) is often used as an indicator of the partitioning behavior of substances. As an equilibrium partitioning coefficient, *K_ow_* describes the distribution of a chemical between water-immiscible *n*-octanol, which is representative of lipids and water [94,97]. However, the use of *K_ow_* is inappropriate for most NMs. Firstly, the fundamental definition of an equilibrium partition coefficient, as expressed by the ratio of equilibrium concentrations of the substance in two solvents, depends on the ability of the substance to reach equilibrium based on chemical activity. In this regard, even though ‘*nanoparticle dispersions can be kinetically stable for a long period of time, they do not reach thermodynamic equilibrium and can consequently not be equilibrated with an additional phase*’ [98]. Furthermore, many NMs prefer to partition at the water-octanol interface, especially when the NM is immiscible in any of the two phases [99,100].

As a solution to the challenges with *K_ow_*, a tissue-blood *PC* can be obtained from in vivo studies by measuring tissue/blood levels at steady state [101]. The result of this empirical approach should be termed a tissue ‘distribution coefficient’ (*DC*), to differentiate it from a true thermodynamic partition coefficient. A *DC* should be understood to represent no more than the ratio of the rate of uptake of the NMs into a given tissue to the rate of the efflux of the NMs from that tissue to the blood. The uptake and efflux of NMs in tissues is expected to be governed by many factors, which include the type and nature of the tissue site, binding with proteins, activity of transporters such as scavenger receptors and other factors. As an example, in the PBPK model for QDs, a *DC* was calculated as the ratio of the affinity of QDs to a given tissue over their affinity to blood [6]. *DC* values varied with time depending on instantaneous QD concentrations in the blood and the tissues, as well as the microenvironment at the tissue site, which was governed by many factors including gaps of the capillary vessels, the topography of the tissue site, possible binding with proteins and/or receptors and a variety of cellular processes. For this reason, for a time period, t_1_ to t_2_, the *DC* was defined as the area under the curve (AUC) of QD concentration in tissue divided by the AUC of the QD concentration in blood [6]. Although the *DC* appears to be a viable alternative to the use of equilibrium partitioning coefficients, this approach, may however, require information on the affinity of NMs to various media and tissues, information that is currently not available for many NMs. *DCs* have been used in PBPK models for various NMs including Au [56], Ag [10] and ZnO [23], which utilized time-varying tissue *PCs* for ZnO described by the Hill function [23].

It needs to be pointed out also that the approach followed for determining *PC*s appear not to be provided in some models such as in PBPK models for gold [15,83], silver [10], 99m-Technetium-labelled carbon [9] and TiO_2_ [17] NPs. Partitioning may be affected by many processes including corona formation, aggregation and agglomeration. The presence or absence of a corona can cause differences in biodistribution and biological outcomes [86]. In one animal model, corona formation may be implicitly included in partitioning by the use of empirically-determined organ-specific and time-dependent partition coefficients as utilized by Chen et al. [23]. However, differences in protein binding to NMs among interspecies makes direct extrapolation of NM biodistribution data across species challenging. In that regard, efforts have been made towards the development of mathematical formulae that can be used to predict the highly complex and dynamic process of corona formation.

Dell’Orco et al. [102], as well as Sahneh et al. [103], developed mathematical models that can predict the time-dependent evolution and equilibrium compositions of corona. Species-specific corona formation kinetics derived from such calculations can be incorporated into PBPK models for animal-to-human model extrapolation [67]. Nevertheless, the inclusion of a quantitative metric for corona formation in PBPK models remains a challenge [104]. The effects of aggregation and agglomeration in PBPK models are presented later in the paper under metabolism.

#### 3.2.3. The Mononuclear Phagocyte system

In the vascular system, NMs are often almost immediately opsonized and distributed to the MPS, where they are engulfed by phagocytic cells [105,106]. The MPS (reticuloendothelial system, RES) comprise of a group of cells, including macrophages, Kupffer cells in the liver, reticular cells in the lymph nodes, bone marrow and spleen, as well as fixed macrophages of various connective tissues, that have the ability to ingest particles [5]. Predominant deposition of stable PAA NPs of the MPS in organs could be demonstrated in the PBPK by Wenger et al. [107]. The dependence of biodistribution on the rate of uptake and release of NPs by phagocytic cells in target organs of QDs was also demonstrated in the PBPK model for QDs, following intravenous administration [8]. Similarly, in the lungs, phagocytic uptake is the main mechanism for the removal of insoluble NMs, involving intravascular, interstitial, pleural and surface macrophages [108], which complement mucociliary transport of deposited NMs from nasal and tracheobronchial airways to the GI-tract [109].

Cells of the MPS rapidly capture NMs until their saturation [110]. These cells constitute a major reservoir of NMs, holding as much as 83% of NMs [24]. Phagocytosed NMs are exposed to various enzymes, reactive oxygen species (ROS) and acidic environments. With some exceptions, such as PBPK models for TiO_2_ NPs [19] and the anticancer agent SNX-2112 [26], recent PBPK models generally included phagocytosis by the MPS system, while older PBPK models did not [6,10,25]. Those examples of models that included the MPS [8,11,17,22,24,34,56,75] divided each of the selected compartments/organs into sub-compartments of blood, tissue and phagocytic cells of the MPS. Critical phagocytosis parameters included in the model were rate of uptake by phagocytic cells, uptake capacity per phagocytic cell, desorption rate of NMs from MPS to tissue as well as number of phagocytic cells (per gram) in various tissues. The MPS saturation level is organ- specific, which indicates the differences in the density of MPS as well as the uptake capacity for the various types of MPS in different organs [24]. The uptake rate should decrease as saturation is approached as characterized by the MPS uptake capacity per unit weight [16,24]. This uptake rate has sometimes been reported to be accurately described by the Hill function (Equation (6)):

(6) $$k_{\mathrm{up}}=\frac{K_{\max}T^{n}}{K_{50}^{n}+T^{n}},$$

where k_up_ is the uptake rate constant by phagocytic cells, T is the time, K_max_ is the maximum uptake rate constant, K_50_ is the time to reach half of K_max_ and n is the Hill coefficient [8]. Deng et al. [16] explored the role of endocytosis in PBPK modelling based on a more complex set of equations, whereas Silva et al. [78] used a mouse cell to estimate MPS uptake for superparamagnetic iron oxide nanoparticles (SPIONS).

There are differences among the macrophages of the MPS in various tissues in terms of the density and kinetic parameters, such as rate of production and rate of migration [106]. However, in some PBPK models all phagocytizing cells are assumed to be similar [22,34], while in other models they are assumed to be different [15,56]. Furthermore, the MPS can be stimulated or suppressed by repeated exposure depending on the dose, dosing frequency and dosing duration [82]. There is a need to consider these factors when incorporating the MPS in PBPK models.

In addition to the MPS, the lymphatic system as a whole, including the lymph nodes, is a major target for NM accumulation [7]. Therefore, exclusion of the lymphatic system is likely to result in significant error in the simulation of distribution of NMs. However, the mathematical description of the entire lymphatic system, which permeates throughout the entire body, is very complex and incorporating it into PBPK models can result in a very complicated model [5]. Consequently, to create a parsimonious model and avoid over-complexity, the lymph system is typically not included in most PBPK models for NMs. Another challenge involves the visualization and sampling of lymph nodes in order to assess trapped NMs [22]. This notwithstanding, in addition to the division of each compartment into sub-compartments of blood, tissue and the MPS, the lymphatic system appears to have been included in the PBPK model for Au NPs by Aborig et al. [15].

The NMs that escape the MPS system and renal clearance (discussed below) are distributed to non-MPS tissues. In that regard, many pharmaceutical NMs are deliberately designed with neutral, hydrophilic or zwitterionic polymers to reduce opsonization and recognition by macrophages and subsequent sequestration by the MPS. This results in higher circulation half-times [53].

Biodistribution and accumulation of NMs have also been shown to depend on the route of exposure [111,112]. Consequently, most PBPK for NMs tend to focus on one route of administration, while only few PBPK models have been developed for multiple routes. In that regard, Bachler et al. [11] developed a model for dermal, oral and inhalational exposure to silver ions and Ag NPs. With proper use of uptake rates for various routes of exposure, these multi-route models could be especially useful for inter-route comparisons.

In addition to being route-specific, most PBPK models tend to be developed for a single type of NM. However, Carlander et al. [75] successfully applied a PBPK model for pegylated polyacrylamide NPs to uncoated polyacrylamide, gold and titanium dioxide NPs, with only changes in NP-specific parameters. Furthermore, Bachler et al. [83] utilized a PBPK model that was originally developed for TiO_2_ NPs [17], based on similar assumptions as a PBPK model for Ag NPs [11], to predict the biodistribution of Au NPs. The general applicability of these models to several NMs may imply some similarities in the biokinetics of these NMs.

### 3.3. Metabolism

Similar to chemical compounds, the term metabolism applies very well to enzymatically degradable NMs such as proteins, PLGA and lipid NMs but not to inorganic NMs, which are generally non-degradable. However, the term may also apply very well to enzymatic degradation of surface ligands of inorganic NMs as well as different organic coatings.

For conventional substances, the liver is the main organ that is responsible for the metabolism of most xenobiotics through a large number of enzymes such as monooxygenase, transferases, esterases and epoxide hydrolase [113]. Where enzymatic degradation is applicable, such as the case with most conventional chemical compounds, in vitro metabolic clearance data from human liver microsomes or hepatocytes is often scaled to the in vivo scenarios. This has often been affected by such factors as “metabolism in tissues other than liver, incorrect assumption of rapid equilibrium of drugs between blood and hepatocytes, presence of active transport through the sinusoidal membrane, as well as inter-individual variability” [114].

Grouped as Phase I (functionalization reactions) and phase II reactions (conjugation reactions) [115], metabolic reactions can involve complex enzymatic mechanisms that can result in non-linear kinetics. However, in most cases, metabolism can be described in a PBPK model using a combination of linear and Michaelis-Menton kinetic equations. In the Michaelis-Menten form, the constants V_max_ and K_m_ are obtained from either in vitro or in vivo empirical measurements [116]. An example of an equation for the liver that includes saturable metabolism is shown in Equation (7):

(7) $$\frac{{\mathrm{dA}}_{L}}{\mathrm{dt}}=Q_{L}\;X\;\left(C_{\mathrm{Art}}-\frac{C_{L}}{P_{L}}\right)-V_{\max}X\frac{C_{L}}{P_{L}}/\left(K_{M}+\frac{C_{L}}{P_{L}}\right),$$

where A_L_ is the amount of chemical in the liver (mg), C_Art_ is the concentration of chemical in the arterial blood (mg/L), C_L_ = the concentration of chemical in the liver (mg/L), Q_L_ is the total (arterial plus portal) blood flow to the liver (L/hr), P_L_ is the liver:blood partition coefficient (concentration ratio at equilibrium), V_max_ is the maximum rate of metabolism (mg/hr) and K_M_ is the affinity constant (concentration at half-maximum rate of metabolism) (mg/L).

Metabolic capacity of the liver depends on the enzyme content of the liver and the activity of the relevant enzyme [117]. Due to lack of data, Lin et al. [6] assumed that the disposition of QDs was associated with the first-order rate of metabolism, kf, in the liver that could be explained by a time-dependent Hill equation.

However, as stated earlier, such enzymatic degradation is often not applicable to NMs, although the term does apply very well to enzymatic degradation of surface ligands of inorganic NMs as well as different organic coatings. For example, it has been shown, that lysosomal R-glucosidase could remove a carboxydextran shell from magnetic iron oxide NMs [118]. Nevertheless, this metabolism-initiated-degradation appears not to have been included in the PBPK model for SPIONS [78]. Similarly, although it has been shown that the polymer shell of PEGylated Au NPs can be degraded by proteolytic enzymes [119], Lin et al. [14] could not include this process in their PBPK model because the pharmacokinetics of this process is not well understood.

Metabolism of NMs has sometimes been defined to include any process that alters their physicochemical properties [5]. These processes may include dissolution, aggregation/agglomeration and corona formation. With regard to the former, some inorganic NMs such as Ag and ZnO undergo rapid dissolution, while other inorganic NMs such as Au and TiO_2_ undergo very slow dissolution, to the extent that they are regarded in PBPK models as not to undergo any metabolism [24,56,67]. For most NMs that undergo fast dissolution, the dissolution kinetics of most NMs have not been well characterized mathematically. Therefore, although dissolution is an important process that determines the fate and toxicity of many NMs, it has only been included in some PBPK models and excluded in others. For example, Bachler et al. [11] developed a PBPK model for ionic silver (Ag^2+^) and Ag NPs that empirically accounted for dissolution of the NPs and the subsequent secondary precipitation of the Ag^2+^ ions to silver sulfide (Ag_2_S). The model could successfully predict the biodistribution of Ag^+^ and 15–150 nm Ag NPs. On the other hand, Demin et al. [12] assumed no dissolution for silver NPs in their “mathematical chamber model” for the interorgan distribution and bioaccumulation of the NMs. Despite excluding dissolution, the PBPK model by Lankveld et al. [10] is reported to also have provided an accurate simulation of NPs. The model was developed from experimental data obtained from Inductively Coupled Plasma–Mass Spectrometry (ICP-MS) measurements of Ag^+^ in different organs after digestion of tissues in nitric acid, which, according to the authors, could digest Ag NPs, metallic Ag and Ag ions and not silver precipitations such as silver chloride. Therefore, since digested silver concentrations represented concentrations of free Ag NPs and Ag^+^ ions and not AgCl, the pharmacokinetic kinetic data could not discriminate Ag^+^ in Ag NPs from dissolved or ionic Ag^+^ [120].

Dissolution was also reportedly accounted for in a PBPK model for QDs in mice [6], where metabolism was described by a time-dependent Hill equation. This model was reported to have “excellent predictive capability for the time-dependent kinetic and distributional changes of QDs.” On the other hand, another PBPK model for QDs in mice assumed that no metabolism occurred in any tissues [7]. The perfusion limited PBPK model was reported to also predict the “experimentally observed persistence of QDs in tissues but not early time profiles or different QD biodistribution.” It is important to note that the two PBPK models for QDs were calibrated with pharmacokinetic measurements of Cd^2+^ obtained using ICP-MS. For that reason, both studies could not discriminate Cd^2+^ in particulate QDs from dissolved ionic Cd^2+^ and therefore may not have provided appropriate pharmacokinetic data for QDs.

Aggregation and agglomeration, which relate to the formation of secondary particles composed of primary particles, also alter the physicochemical properties of NMs which ultimately affect their fate and toxicity [113,121,122,123]. In that regard, highly agglomerated multi-walled carbon nanotubes (MWCNTs) were retained in lungs and the liver, where they could not be eliminated in 28 days, in comparison to well-dispersed MWCNTs that remained well dispersed and were more easily eliminated from the circulation through filtration effects of the capillary bed as well as uptake by macrophages [124].

The translation of traditional aggregation/colloidal science to NMs is challenging because of their unique physicochemical properties as well as the complexity of the biological environments [125]. For these reasons, aggregation/agglomeration has not been included in PBPK models [22]. Therefore, there is need for more studies on the mathematical modelling of aggregation of NMs.

### 3.4. Excretion

Excretion refers to the removal of NMs from the living system without decomposition or significant changes in properties from their original form [5]. While the liver and the kidneys are often the major organs responsible for NP excretion through feces and urine, respectively [126], NMs may also be excreted through lungs, breast milk and sweat. However, PBPK models for NMs primarily focus on renal and hepatobiliary clearance [22,24]. Indeed, biliary and urinary excretion rates were shown through sensitivity analyses to be highly influential parameters in PBPK models for a number of NMs including PEGylated Au [14].

The kidney is capable of rapid removal of NMs from the circulatory system, with minimal metabolism. Renal clearance is a process that involves glomerular filtration, tubular secretion and subsequent elimination through urinary excretion [80]. Renal clearance has been shown to be dependent on the size of NPs, where NMs < 5.5 nm can be efficiently and completely eliminated by renal clearance [87], while the renal clearance of NMs with larger size is very slow [127]. Aborig et al. [15] assumed that renal excretion was negligible for Au NPs larger than 10 nm in diameter.

Renal clearance is often included in PBPK models from the vascular compartment of the kidney to the urine compartment [13]. Renal clearance is estimated using renal clearance or urine elimination rates in the kidney compartment, as shown by Equation (8):

(8) $$\frac{{\mathrm{dA}}_{\mathrm{Urinary}\;\mathrm{excretion}}}{\mathrm{dt}}=R_{\mathrm{Urine}}={\;k}_{\mathrm{Urinary}\;\mathrm{excretion}}{\;\times \;\mathrm{CV}}_{K},$$

where R_urine_ is the urinary excretion rate of the NMs in mg/h, k_urine_ is the urinary excretion rate constant of the NMs in L/h and CV_K_ is the concentration of the NMs in the venous blood of the kidneys in mg/L [11,14]. These data are often obtained from the literature. As an example, in their PBPK model for Au NPs, Lin et al. [14] used rates from the study on tissue kinetics of PEG-coated Au NPs by Cho et al. [126]. The excretion rate constant can be described using a first-order elimination from blood (Equation (9)):

(9) $$\frac{{\mathrm{dA}}_{\mathrm{urinary}\;\mathrm{excretion}}}{\mathrm{dt}}={\;k}_{\mathrm{urinary}\;\mathrm{excretion}}{\;\times \;A}_{\mathrm{blood}},$$

where A is the concentration of the NM in blood and t is time [11,78].

The model developed by Lin et al. [14] could adequately describe the biokinetics of Au NPs in mice. The model was later extended to include other species such as pigs [56], where urine excretion rates for the pig sub-model were estimated by visual fitting of the urine data from Fent et al. [128]. On the other hand, the model by Carlander et al. [75] could accurately simulate the biokinetic profile of TiO_2_ NPs after setting urinary excretion of the NPs to zero, in accordance with the observations in the literature [129].

While some authors use Equation (8) for urinary excretion, Bachler et al. [17] hypothesized that the excretion of TiO_2_ NPs occurred via transcapillary pathway and therefore utilized Equation (10) to estimate the urinary excretion rate:

(10) $$k_{\mathrm{trans}-\mathrm{blood}-\mathrm{kidney}}=b_{\mathrm{trans}-\mathrm{constant}-\mathrm{kidney}}\;\times \;\frac{Q_{\mathrm{kidney}-\mathrm{blood}}}{V_{\mathrm{blood}}}$$

where k_trans_blood_organ_ is the translocation rate of nano-TiO_2_ from blood to urine (min^−1^), b_trans-constant-kidney_ is the unitless NM translocation constant for kidneys, representing the permeability of the capillary wall, Q_kidney-blood_ is the flux of blood through the kidney in L/min and V_blood_ is the total blood volume in the body in L.

Urinary excretion is much smaller than biliary excretion because of the pore size of the liver capillaries, which are much larger than the capillary pores of the kidneys [17]. Therefore, for particles that do not undergo appreciable renal clearance, the hepatobiliary system represents the primary route of their excretion [67], involving hepatocytes followed by excretion into the bile [80]. In PBPK modelling, hepatobiliary clearance is presented from the extravascular compartment of liver to a fecal compartment, representing the transport of NMs from liver to gut through bile [13], as shown in Equation (11) (which is similar to Equation (6) above):

(11) $$\frac{{\mathrm{dA}}_{\mathrm{Biliary}\;\mathrm{excretion}}}{\mathrm{dt}}=R_{\mathrm{Bile}}={\;K}_{\mathrm{Bile}}{\;\times \;\mathrm{CV}}_{L},$$

where R_bile_ is the biliary excretion rate of the NMs in mg/h, K_Bile_ is the biliary excretion rate constant of the NMs in L/h and CV_L_ is the concentration of the NMs in the venous blood of the liver in mg/L [11,56].

Biliary excretion rates are often obtained from optimization with in vivo pharmacokinetic data [11,14,15]. Bachler et al. [17] also utilized an equation that is similar to Equation (8) corresponding to the liver. Similar to urinary excretion, Carlander et al. [22] set the fecal excretion of TiO_2_ NPs to zero as supported by in vivo studies, which indicate minimal fecal excretion for TiO_2_ NPs [129]. In the model by Wenger et al. [110] fecal excretion of PAA was also very low, at less than 5% of the administered dose. In contrast to these models, the PBPK model by Li et al. [21] indicated some significant fecal excretion of CeO_2_ NPs. However, these NPs were said to predominantly originate from the GI tract as a result of mucociliary clearance and not from biliary excretion, an observation that was supported by the high concentrations of CeO_2_ NPs in the GI tract as compared to those in the liver. Therefore, there is need to understand the contribution of each one of these pathways to the excretion of NMs. In that regard, through the measurement of radioactivity, Péry et al. [9] established an equation (Equation (12)) for the fraction of inhaled carbon NPs that were translocated to the stomach:

(12) $$Q_{\mathrm{stom}}=\;S\_{\mathrm{fQ}}_{\mathrm{lung}}$$

where Q_stom_ is the quantity of NMs in the stomach, Q_lung_ is the quantity of NM in the lung and S_f is the fraction of the inhaled particles that is swallowed. Equation (12) was empirically determined for carbon NPs and may not be applicable to other NMs. Therefore, in order to assess the fraction of NMs in feces that result from mucociliary clearance there is need to determine similar equations for other NMs.

In addition to size, biliary excretion of NMs has been shown to be affected by functionalization [130]. However, there is limited knowledge about the effect of these properties as well as other properties on biliary excretion. Therefore, development of appropriate in vitro and in vivo assays for the assessment of NM excretion is needed for the development of more accurate PBPK models [67].

## 4. Model Evaluation and Validation

In addition to a demonstration of model plausibility with regards to physiology, it is important to show that simulation results are in agreement with observed data [30]. In that regard, PBPK models are evaluated in sensitivity and validation analyses. Sensitivity analysis are undertaken to identify parameters of highest concern, that is the parameters that are known with least certainty but have large influence on model performance [30]. Sensitivity analysis can identify the parameters that substantially influence model outputs [131,132]. Parameters are considered sensitive if a modest change to the parameter results in a noticeable change in a simulation output or “when a disturbance shifts the model prediction beyond the experimental error” [28].

For model parameters with high normalized sensitivity coefficients, the relative change in output is more than the relative change in the parameter; that is, small parameter changes result in large changes in model output. Therefore, improvements in the knowledge of those parameters can increase the model accuracy [36].

For deterministic PBPK models, sensitivity analysis can be grouped into two categories, including *global approaches,* which calculate the contribution of a parameter among all possible input parameters, as well as the *local* or *one-at-a-time (OAT)* approaches that consider sensitivities around a specific set of input parameters [133]. Application of the former approach in a PBPK model involves the perturbation of parameters for compartments (e.g., organs and tissues), blood flow rates, metabolic parameters, permeability coefficients and *PCs* within plausible ranges. Subsequently, the contribution of any single parameter or interactions of multiple parameters to the model output is measured to give an indication of the overall relative importance of all model parameters. The latter approach appears to be the most widely used approach in PBPK models for NMs because it is fairly rapid and simple to implement although it can result in misleading results if there are substantial interactions among a number of parameters [133]. In this approach, sensitivity analyses are evaluated through the use of normalized sensitivity coefficients that are calculated by dividing the percent change in the prediction of interest induced by the percent change (usually 1–10%) made to a single model parameter [131]. For example, (Equation (13)):

(13) Relative sensitivity=dAUCiAUCidpjpj ,

where AUCi represents area under the curve for compartment i, and p_j_ represents the parameter i. Similarly, Chen et al. [23] calculated sensitivity ratios (SRs) by increasing PCs and excretion and elimination rates by 10%, using the following equation (Equation (14)):

(14) SR=ΔCCoΔxxo,

where ΔC is the difference between the resulting and original (Co) predicted concentration values and Δx is the difference between the resulting and initial (xo) parameter values. Instead of a change of 10% in a parameter, other authors have used a change of 1% [21,83] and therefore there is a need for consistency in this regard.

Li et al. [24] found that the model sensitivity to physiological parameters was size-dependent. Furthermore, the non-linearity resulting from saturation of MPS implies that the concentration of NMs in tissues does not increase in proportion to the dose. This results in sensitivity coefficients that vary with the dose [22]. As discussed earlier, analysis of uncertainty based on a frequentist approach only takes into consideration the data collected in the present study. The plausibility of such results is often a cause for concern and as such they need to be assessed in the light of previous studies [42]. The uncertainties can be reduced through the use of the Bayesian and probabilistic techniques [44,134]. In those studies, sensitive adjustable model parameters were identified by using posterior parameter values derived from Markov chain Monte Carlo (MCMC) analysis. Nevertheless, application of the Bayesian approach to the PBPK modelling of some NMs may be limited by the scarcity of toxicokinetic data.

Confidence in the predictive performance of the model is high when the mechanisms of the underlying processes are identified, the associated parameters are determined and the model is validated against experimental data [135]. Model validation, defined as the process by which the reliability and relevance of a particular model is established [136], assesses the ability of the model to predict the toxicokinetic behavior of the chemical under consideration, preferably using “data that was not used in the development of the model and the estimation of its parameters” [101]. Comparisons between measured and predicted data can be made for typical pharmacokinetic parameters such as maximum plasma concentrations (C_max_), time to reach maximum plasma concentration (*t_max_*), AUC and half-times (*t_1/2_s*) [137]. Sweeney et al. [134] calculated a discrepancy index (i.e., the maximum of the predicted value/experimental value or experimental value/predicted value) for each experimental data point, where the discrepancy index of 1 indicated a perfect agreement between the predicted and experimental data.

In some PBPK models the deviation from the 1:1 line between measured and predicted values was used to evaluate the models [34,83]. Furthermore, some authors used the coefficient of determination, R^2^, to indicate the level of agreement between measured and predicted values [8,22,23,24,34,93]. R^2^ is a well-known statistic that describes the proportion of variance in the outcome variable that is explained by the independent variable. In other words, the statistic describes how well the model fits the data, where values close to 1 imply an almost perfect relationship. Nevertheless, R^2^, along with its related statistic correlation coefficient (r), have many limitations and their use for assessing agreement between two methods has been discouraged [138,139]. PBPK modelers are therefore urged to explore and use other methods, such as the root mean square error (RMSE), modelling efficiency (ME) [140], the Nash-Sutcliffe efficiency (NSE) [141], the Root Mean Deviation (RMD) [140], as well as the use of precision of estimated limits of agreement [138]. Although these quantitative tests of goodness of fit are very useful, it is also important to assess the ability of the model to predict the general trend or shape of the time-course data, including peaks and troughs [33,142].

Model performance is considered adequate if the predicted values are within a factor of two of experimental data [136]. A number of models have been reported to offer predictive ability within this limit, including but not limited to, models by Lin et al. [11,56]. During the validation process it is important to consider that both experimental and simulation data are subject to uncertainty. For PBPK models for NMs, a saturable uptake of NMs in MPS means that as the MPS approach saturation at high doses, tissue levels will be less than proportional to the dose. Therefore, models require validation against non-saturating (low) as well as saturating (high) doses for which unfortunately data is not available [22]. Similar to the sensitivity analysis, the validation process can be performed using deterministic or probabilistic approaches as well as frequentist or Bayesian approaches.

Although the challenges associated with the development of PBPK models are well-documented [67], a number of PBPK models have been developed in the past 10 years (Table 1). Most of the models have been developed in rodents, with only very few developed for humans. Furthermore, most of the models have been developed for one route of exposure, with intravenous injection being the most common route. These models have required varying amounts and types of input data. On one hand are top-down models, such as the model by Lankveld et al. [10], that are developed by empirically fitting the model to the kinetics data of the NMs in blood and various organs, simultaneously. These models use little or no mechanistic calculations and thus require the least amount of input data. These models require mostly kinetic data for model calibration and curve fitting. On the other hand, there are bottom–up models, such as models by Chen et al. [23] and Lin et al. [56], that are developed more mechanistically by mathematically describing the relationships between some variables based on underlying principles. These models can either be perfusion-limited or permeability-limited. In addition to blood flow values, *PC*, *V_t_* as well as the concentration of NMs in blood that are required in perfusion limited models, permeability models will require permeability coefficients for permeation into various tissues.

Both perfusion- and permeability-limited models will require uptake rates following administration, as well as metabolism (if any) and excretion. These are often empirically derived but more recent models have included time-dependent endocytosis that can be described by the Hill equation or the Michaelis-Menten equation. In most PBPK models, metabolism is taken to be negligible except for the NMs with significant dissolution rates such as Ag and ZnO. Therefore, PBPK models for NMs with significant dissolution will require dissolution rates or kinetic constants among input data. As Table 1 shows, some of the PBPK models are reported to possess high predictive capability, within the limits of acceptability as stipulated by the World Health Organisation (WHO) [33].

As the parameters required for PBPK modelling are high in number and relatively difficult to obtain, their estimation using computational approaches is advantageous. An approach for the estimation of adipose/blood partition coefficients through QSAR for 67 environmental chemicals, which was also used for gap filling of the logP_(adipose/blood)_ data for 513 chemicals from the US EPA, was reported by Jean et al. [143]. Quantitative property-property relationships (QPPRs) have been applied to the high-throughput prediction of internal dose of inhaled organic chemicals in PBPK models [144], while physiologically based toxicokinetic (PBTK) model parameters have been calculated by Sarigiannis et al. [145] and Savvateeva et al. [146]. Research specific to parameters of nanomaterial PBPK models is less common but increasing—a method combining an artificial intelligence-based cell simulation and a calibrated fluorescence assay that quantifies rate constant for biological interactions between NMs and individual cells is proposed by Price and Gesquiere [147]. A more complete is presented in the multiscale modelling approach for drug mechanism and safety that has been proposed for chemicals by Zhang et al. [148] and that could serve as a guide for integrating models at different scales—at the molecular, cellular and omics and organ and system level.

## 5. Application of PBPK Models to Hazard Assessment of Nanomaterials

While most PBPK models have been used to estimate the biodistribution of NMs, Laomettachit et al. [20] examined the toxicity of TiO_2_ NPs on human liver in a two-step approach that combined a PBPK model and a cell-response model, where the latter model was used to predict liver cell viability and cell death resulting from accumulated TiO_2_ NPs. This approach, which can directly assess the risks of NM exposure, is only possible in cases where the pharmacodynamics (i.e., concentration–effect relationships) are well understood. Indeed, the approach is part of physiology-based pharmacodynamic (PBPD) modelling, which has been used extensively in drug discovery. PBPD models extend PBPK concepts to quantitatively characterize the effects (expressed as change in biological function) of chemical exposure (expressed as concentration at the target site) [149]. Such PBPD models can be developed from existing toxicodynamic compartmental models such as the toxicodynamic model by Mukherjee et al. [150], which predicts effects of inhalation of Ag and carbon NPs on biological and mechanical responses in the lung. The model has some level of complexity including the considerations of chemical properties of the NPs such as size, composition and surface zeta potential, as well as properties of the cellular environment such as cell diameter, fluid velocity in the vicinity and cell packing density.

The unique challenge in evaluating potential adverse effects of nanomaterial exposures arises from the role of their physicochemical properties in determining their biological impacts as well as kinetic behavior in the body. The size, chemical composition, surface properties, solubility, shape and aggregation are all properties that can potentially modify cellular uptake, interaction with proteins, translocation from portals of entry into the target tissues and possibility of causing tissue injury [151]. These properties also need to be considered when selecting an appropriate internal dose metric for the nanoparticles in the target tissue or cells. Indeed, several findings suggest that toxicity of nanomaterials observed in vivo or in vitro may be correlated with chemical composition/surface activity [152], rather than mass.

Application of PBPK models in the elucidation of adverse effects of NMs require PBPK models that can provide as much information as possible about local physiological concentrations of NMs in relevant organs. A good example is the PBPK model that could describe the differences in the extracellular and intracellular kinetics of the different classes of nickel compounds, including particulate Ni compounds [76]. However, such a model would require more parameters that may not be readily available including density of different types of cells in various organs, intracellular diffusion rate, as well as intracellular and extracellular dissolution rates.

## 6. Application of PBPK Models to Risk Assessment of Nanomaterials

As indicated in the introduction, the application of PBPK models allows the use of internal doses, which are more closely associated with biological responses than the administered dose. In that regard, PBPK models can provide predictions of doses that are relevant to the mode of action (MOA) of chemicals. For these reasons, PBPK models that are intended to be used in risk assessment should adequately simulate the relevant dose metrics for the anticipated exposures, exposure route, dose ranges and species as well as critical life stages [136]. Consequently, there are challenges that are associated with the application of PBPK models in risk assessment, especially lack of confidence in the PBPK models for which no tissue/plasma concentration data exist for model evaluation [153]. For these reasons, in addition to a demonstration of predictive capability and biological plausibility, PBPK models used in risk assessment should be developed and evaluated in the species and life stages that are of relevance to the risk assessment and for exposure doses, routes and durations that correspond to anticipated human exposures.

There have been attempts to apply a number of PBPK models in the risk assessment of NMs. Bachler et al. [17] used a PBPK model in the dietary risk assessment of nano-TiO_2_ for the German population, where dermal and/or the inhalation routes could also be easily added to the model. The risk assessment indicates that the risk from the ingestion of nano-TiO_2_ through foods and beverages, drugs and dietary supplements as well as toothpastes for the German population is small.

Bachler et al. [11] applied a PBPK model in the risk assessments of Ag NPs in many scenarios following dietary exposure, oral uptake of Ag NPs released from food boxes, dermal uptake of Ag NPs released from T-shirts, oral uptake of emitted Ag NPs from a throat spray and occupational exposure. The model indicated that the risk of adverse health effects from exposure to Ag NPs in consumer products is very small, except after the usage of the colloidal silver throat spray. Most importantly, the model could show that occupational exposure to Ag NPs could induce adverse health effects even for exposures that were below the legal limit for Ag NPs in many countries. The model could show that inhalation was the most critical route of exposure. However, the risk assessment was hampered by large uncertainties and limited knowledge of Ag NP occupational exposure and release rates from consumer products as well as Ag NP absorption rates into the systemic blood circulation. Indeed, large uncertainties and lack of information are not only limited to Ag NPs but also all NMs. In that regard, the Bayesian approach has been used to improve risk assessment of NMs [9,134].

## 7. Overall Assessment and Conclusions

Early PBPK models applied for NMs tended to be more empirical and top-down, while more recent models are becoming more mechanistic. Models developed recently are often based on permeability-limited processes and not perfusion-limited processes, which are more suitable for small molecules. On the other hand, permeability-limited models require use of permeability values, which are currently very difficult to obtain. For these reasons, there are differences in the use of permeability values, where some models utilize one permeability value for all organs, often with the exception of the brain, while others use organ-specific values. A permeability of zero is often used for the brain although it has been shown that NMs can circumvent the BBB through the sensory neuronal systems and the olfactory bulb [92]. The neural transport of NMs is not well characterized. Any of these descriptions, which assume transport is driven simply by differences in chemical activity, are clearly inappropriate for nanoparticles, where very different processes associated with receptor mediated transport and endocytosis are more likely to drive uptake and clearance [74].

The development of more accurate PBPK models requires a better understanding of uptake processes for NMs and how the processes depend on the route and physicochemical properties of NMs. Little is known, for example, on the fraction of NMs deposited in the upper respiratory tract that is cleared by coughing, swallowing and the mucociliary escalator, despite the use of lung dosimetry models. The recent use of alveolar epithelial cellular monolayers at the ALI for PBPK modelling [83] will undoubtedly provide information on translocation kinetics of NMs across the lung epithelium. Furthermore, there is a poor understanding of how different types of NMs behave in the GI tract as they encounter food matrices and biopolymers. In that regard, there is need to incorporate in PBPK models for NMs results from recent NM dissolution systems that utilize a cascade of digestive fluids encountered as NMs pass through the GI tract [154]. In the GI tract, some NMs may be trapped in mucus and expelled through feces, while others migrate through the epithelium to reach the systemic circulation [63]. NMs that evade absorption are combined with those that are absorbed and undergo hepatobiliary excretion [155]. Kinetics data are needed for mechanistic inclusion of these processes in PBPK models. Paramount in this regard are the fractions of NMs cleared from the upper respiratory tract by coughing, swallowing and the mucociliary escalator, as well as the pharmacokinetic behavior of NMs in the presence of food matrices and biopolymers in the GI tract.

NMs that enter the circulatory system are subject to corona formation. As discussed earlier, there are interspecies differences in protein corona formation that make direct extrapolation of NM biodistribution data across species really challenging. Therefore, there is need for more efforts on the development of mathematical models that can mechanistically explain this process in various species and biological environments. In the circulatory system, NMs also encounter the MPS, which is involved in the biodistribution of NMs. The MPS are increasingly being included in PBPK models for NMs although their phagocytosis kinetics for NMs is not well understood. As discussed earlier, some PBPK models utilize the same MPS phagocytosis rate for all organs, although there is heterogeneity among the macrophages of the MPS [22,34], while other models utilize different organ-specific MPS phagocytosis rates [15,56]. More accurate information on this process is required since sensitivity analyses have shown that the MPS uptake capacity can be one of the most influential parameters in PBPK models [24].

The analysis of PBPK models presented earlier showed that dissolution, which is a key elimination process for some NMs, is not well understood and presented in PBPK models. There is a need for more studies on dissolution kinetics of various NMs including kinetics of the formation of secondary particles such as Ag_2_S. Dissolution is strongly affected by pH with some NMs undergoing dissolution in neutral extracellular fluids, while others undergo dissolution in low-pH intracellular fluids [156]. Development of more accurate PBPK models will require more data on dissolution of NMs in these different environments, focusing particularly dissolution rates, rate constants, orders of reaction, half-times and life-times [157]. Modelling elimination of NMs will also require data on rates of biliary and renal excretion for various NMs, as well as their dependence on various physiochemical factors, particularly size and functionalization.

The developing fetus and infant represent important potentially vulnerable subpopulation of humans. Due to dynamic changes occurring during early life development and growth, normal barrier function to NM exposure may be different compared to adults. PBPK models can provide a means to predict fetal and neonatal target tissue exposures and compare them to those of adults. The key data needs for this modelling are transport characteristics across maternal barriers such as the placenta and translocation into milk at mammary gland cells. 

The PBPK models for NMs published to date have primarily focused on large-scale pharmacokinetics, such as lung deposition and clearance and systemic distribution. Since basic information is generally lacking regarding cellular uptake processes, facilitated transport across epithelial barriers, mechanisms of intracellular sequestration and mechanisms of cellular clearance, these studies have assumed that nanoparticles would behave similarly to chemicals that distribute into tissues on the basis of thermodynamic partitioning or employed simple empirical descriptions for the tissue distribution processes. However, a PBPK modelling study of quantum dots concluded that it was impossible to capture the observed kinetics using the traditional assumption of tissue partitioning and suggested a more detailed description of cellular kinetics would be required beyond simple partitioning [7]. Thus, an important area of research is to extend PBPK models of NMs to describe intracellular kinetics in order to quantify the disposition of the NMs in the target cell. Such PBPK models could be used to investigate observed differences in the cellular kinetics of different NMs.

A cellular dosimetry model for nickel particles [76] demonstrates an approach that could, in principle, be applied for modelling intracellular NM kinetics. To describe the cellular disposition of nickel particulate, it was necessary to model the extracellular and intracellular kinetics of nickel compounds with different solubilities. When coupled with a lung deposition model, the resulting cellular dosimetry model was able to estimate the cellular exposure to nickel resulting from inhalation of different forms of nickel. Given the importance of physicochemical properties in determining cellular uptake of NMs at the biological barrier, evaluation of the major determinants for the observed differences in the extracellular and intracellular kinetics of NMs may also be possible. Recent advances in imaging technology make it possible to link the cellular uptake and localization of NMs with observed cytotoxicity [36]. The resulting cellular dosimetry model can then be integrated with lung deposition/clearance modelling in conjunction with a whole body PBPK model based on the newly collected data on systemic distribution/clearance of NMs. The use of such an integrated PBPK model would allow for more biologically based risk estimates for exposures to NMs.

Few PBPK models for NMs have been published in the literature with the relevant computer codes [6,8,14,45]. However, there appears to be no PBPK model for NMs in a publicly available software format. This lack of computer code or software hampers independent verification of the models as well as the development of the field since the models are not easily or immediately usable by other scientists. Indeed, lack of appropriate modelling expertise and experience has been cited as one of the challenges that hinders the application of PBPK models in public health decision-making [153].

An efficient way to make PBPK models available to the community for testing on new data is provided by the Jaqpot platform [158], which offers nano-aware PBPK modelling in the NanoCommons infrastructure (https://infrastructure.nanocommons.eu), a functionality that will be integrated into the NanoSolveIT e-Infrastructure (https://nanosolveit.eu). An example was presented for nano PBPK model for polyethylene glycol-coated polyacrylamide (PAA-peg) nanoparticles on rat [24]. Users can obtain predictions from the model by providing new data on Jaqpot user interface or upload a comma-separated values (CSV) file with the relevant values. Lack of appropriate modelling expertise and experience has been cited as one of the challenges that hinders the application of PBPK models in public health decision-making [153].

Therefore, notable in this regard is the translation of a number of PBPK models, including a PBPK model for Au NMs, from acslX (a now defunct software) into a number of computer languages including Berkeley Madonna, MATrix LABoratory (MATLAB) and R Language [159]. In addition, the Exposure Related Dose Estimating Model (ERDEM) developed by the U.S. EPA [160] has been utilized to develop PBPK models for a number of molecular substances [161,162,163]. However, there appears to be no application of the program to NMs in the literature. A similar generalized model was developed by the Canadian Centre for Environmental Modelling and Chemistry [164]. The model requires chemical properties such as molar mass, *K_ow_* and *K_aw_* and may not applicable to NMs.

In conclusion, there have been great advances in the development and application of PBPK models for NMs. Furthermore, there have been attempts to apply several models in the risk assessment of NMs. In that regard, PBPD models, which are an extension of PBPK models, have been used to quantitatively characterize biological effects following exposure to some NMs. Some of the PBPK models are validated and show excellent agreement between predicted and observed data. However, development of even more accurate PBPK/D models hinges on the greater understanding of the pharmacokinetics and biological effects of NMs in humans and other species.

## Figures and Tables

**Table 1:** Summary of existing physiologically based pharmacokinetic (PBPK) models for nanomaterials (NMs).

NM Type	NM Abbreviation	In vitro and/or In Vivo Model Used for PBPK Modelling	Route	Key Feature/Result	Model Validation	Reference
Bioconjugates, inorganic NPs and metal-oxide NPs	PAA, PEGylated and non-PEGylated (size not indicated)	Rat	Intravenous injection	Developed entirely from in vivo kinetic data	Validation not indicated	[110]
	31 nm PAA and PAA-PEG	Rat	Intravenous injection	Developed from in vivo kinetic data; did not include MPS as an organ	Validation not indicated	[107]
	20, 31, 80, 114, 319 nm PEG-PAA, PAA, breviscapine-loaded poly(D, L-lactic acid) (BVP-PLA)	Rat	Intravenous injection	The model included MPS where all MPS had the same efficacy and saturation level, independent of their location	Predictions fitted the experimental data relatively well, with R^2^ values ranging from 0.707 to 0.994	[34]
	35 nm PEG-PAA	Rat	Intravenous injection	Diffusion-limited model, different MPS uptake capacities were used for each organ	Prediction valued matched measured data (R^2^ = 0.97)	[24]
	35 nm PEG-Au	Mouse	Intravenous injection	Permeability-limited model preferred over flow-limited; included MPS	Model predictions compared very well with experimental data, within a factor of two	[14]
	13–100 nm PEG- gum arabic or citrate-Au	Mouse, rat, pig and human	Intravenous injection	A permeability-limited model for several species using a general approach for endocytosis	Simulation results were within a factor of two of independent experimentaldata	[56]
	100 nm Dexamethasone-encapsulated nanoparticles(Dex-NPs)	Mouse	Intravenous injection	Perfusion-limited model where absorption of the NMs in the organ was modeled via equilibrium partitioning	Simulation results were consistent with previously published in vivo data	[28]
	13,15,20,40,80,100 nm Au-PEG	Miceand humans	Intravenous injection	The model explored extensively the role of endocytosis in PBPK using a set of equations	The model predicted NP distribution very well in both mice and humans	[16]
	Molecular imaging NPs (MINPs) based on peptide nucleic acids (Size not indicated)	Mice	Intravenous injection	A permeability-limited model that did not include MPS	Model predictions compared well with experimental data	[27]
	203 nm Nano SNX-2112 (anticancer agent)	Rat	Intravenous injection	Pharmacokinetic of the nanoform was similar to that of the nanoparticulate form due to rapid dissolution	Model predictions compared very well with experimental data	[26]
	31 nm PEG-PAA, 31 nm uncoated PAA,13, 56 nm Au and 63 nm TiO_2_	Rat	Intravenous injection	The model included the MPS, using both flow and permeability-limited processes	The model reported to explain 97% of the observed variation in biokinetics of PAA	[75]
Inorganic NPs	18.5 nm QDs	Mouse	Intravenous injection	The model made use of time-dependent PCs	Model reported to have excellent predictive capability	[6]
	QDs (13 nm QD705, 12 nm QD525, 21 nm QD800, 37 nm QD621, 7–25 nm QD-LM, 80 nm QD-BSA)	Mice and Rats	Intravenous and intradermal injection	Perfusion-limited model, with fixed PCs and assuming no elimination or metabolism occurred in any tissues	The model could not adequately describe the complex biodistribution exhibited bydifferent QDs	[7]
	3.5 nm QDs	Mouse	Intravenous injection	The model used permeability-limited processes using organ-specific PCs	Predicted data matched independent experimental data	[8]
	15 to 20 nm Iridium and Ag	Rat	Endotracheal instillation and inhalation	Model included the lymph system and olfactory system.	Model not calibrated but calibration data fitted very well with experimental data,	[93]
	20, 80 and 110 nm Ag	Rat	Intravenous injection	The model did not explicitly include dissolution of Ag	Model predictions compared very well with experimental data, within a factor of two	[10]
	15–150 nm Ag	Rat and human	Dermal, oral and inhalation	The model combined ionic and nanoparticulate PBPK sub-models	Validated by comparing with experimental values	[11]
	25 nm Au	Mouse	Intraperitoneal injection	The model included the MPS and the whole lymphatic system	Model predictions were within 1.48-fold of the observed values in all organs	[15]
	2, 7, 18, 46 and 80 nm Au	In vitro alveolar epithelial cellular cultures	The ALI for PBPK modelling	Translocation kinetics of Au NPs across the lung epithelium determined using ALI	Translocation kinetics were adequately predicted for mice after inhalation exposure, while for rats after intratracheal instillation, the translocation was slightly overestimated	[83]
Metal-Oxide NPs	10 nm and 71 nm ZnO	Mice	Intravenous, inhalation and oral exposure	Dissolution of ZnO was not specifically included. The model used time-dependent PCs	Simulation of ZnO NPs only fitted the experimental data after replacing PCs of ZnO NPs with those for Zn(NO_3_)_2_	[23]
	25 and 90 nm CeO_2_	Rats	Inhalation	The model used both flow- and permeability-limited processes using time-dependent PCs	The model successfully predicted the kinetics of CeO_2_ NPs	[21]
	21 nm Superparamagnetic nanoparticles (SPIONs): magnetite (Fe_3_O_4_) and maghemite (γ-Fe_2_O_3_), SPIONs	Mice	intravenously in a single dose	Novel in vitro experimental data describing uptake of SPIONs in murine macrophage cell line and primary human monocyte-derived macrophages were integrated into this computational approach.	The PBPK model generated was compared against in vivo results and showed to be effective in the prediction of the SPION distribution.	[78]
	20 nm TiO_2_	Rats	Intravenous injection	The model used combined a PBPK model and a cell-response model to predict liver cell viability and cell death	Not validated	[18,20]
	15–150 nm TiO_2_	Human	Oral administration	Permeability-limited model that did included the MPS	Evaluated by comparing simulated organ levels to experimentally assessed organ levels of independent in vivo studies	[17]
	25 nm TiO_2_	Rats	Intravenous injection	Perfusion-limited model that did not include the MPS	The PBPK model was outperformed by a simple compartmentalmodel	[19]
	5, 15, 30, 55 nm CeO_2_ with citrate coating	Humans	Intravenous injection, inhalation, intratracheal instillation and oral exposure	The model included the MPS, using both flow and permeability-limited processes	The model adequately described CeO_2_ biokinetics in various tissues for the 5 nm ceria as well as for the 30 nm ceria in liver and spleen	[22]
Carbon-based-NPs	5–10 nm Radiolabeled CNTs	Humans	Inhalation	Developed from time-courses of radioactivity in various organs, with one common fixed PC	Prediction results were consistent with previously published in vivo data	[9]

## Abbreviations

ADME	Absorption, distribution, metabolism and elimination
Ag	Silver
Ag_2_S	Silver sulfide
ALI	Air-liquid interface
Au	Gold
AUC	Area under the curve
BBB	Blood–brain barrier
BMD	Benchmark dose
BMC	Benchmark concentration
CeO_2_	Cerium dioxide
CSV	Comma-separated values
*DC*	Distribution coefficient
ERDEM	Exposure Related Dose Estimating Model
GI	Gastrointestinal
ICP-MS	Inductively Coupled Plasma–Mass Spectrometry
ICRP	International Commission on Radiological Protection
IVIVE	In vitro to in vivo extrapolation
*K_ow_*	Distribution coefficient
LOAEL	Lowest-observed-adverse-effect level
MATLAB	MATrix LABoratory
MCMC	Markov chain Monte Carlo
ME	Modelling efficiency
MOA	Mode of action
MPPD	Multiple-Path Particle Dosimetry
MPS	Mononuclear phagocyte system
MWCNTs	Multi-walled carbon nanotubes
NMs	Nanomaterials
NOAEL	No-observed-adverse-effect level
NSE	Nash-Sutcliffe efficiency
PAA	Polyacrylamide
PEG	Polyethylene glycol
PBPD	Physiology-based pharmacodynamic
PBPK	Physiologically-based pharmacokinetic
*PCs*	Partition coefficients
*P_t_*	Permeability
PLGA	Poly(lactic-co-glycolic) acid
QDs	Quantum dots
QPPRs	Quantitative property-property relationships
QSPR	Quantitative structure/property relationship
RES	Reticuloendothelial system
ROS	Reactive oxygen species
RMSE	Root mean square error
SPIONS	Superparamagnetic iron oxide nanoparticles
TiO_2_	Titanium dioxide
ZnO	Zinc oxide
